# Training for Defense? From Stochastic Traits to Synchrony in Giant Honey Bees (*Apis dorsata*)

**DOI:** 10.3390/insects3030833

**Published:** 2012-08-30

**Authors:** Frank Weihmann, Thomas Hoetzl, Gerald Kastberger

**Affiliations:** Department of Zoology, University of Graz, Graz 8010, Austria; E-Mails: thohoe@aon.at (T.H.); gerald.kastberger@uni-graz.at (G.K.)

**Keywords:** Giant Honey Bee, *Apis dorsata*, social insects, flickering behavior, recruitment for defense, communication, flickering-shimmering transition hypothesis

## Abstract

In Giant Honey Bees, abdomen flipping happens in a variety of contexts. It can be either synchronous or cascaded, such as in the collective defense traits of shimmering and rearing-up, or it can happen as single-agent behavior. Abdomen flipping is also involved in flickering behavior, which occurs regularly under quiescent colony state displaying singular or collective traits, with stochastic, and (semi-) synchronized properties. It presumably acts via visual, mechanoceptive, and pheromonal pathways and its goals are still unknown. This study questions whether flickering is preliminary to shimmering which is subject of the *fs* (*flickering-shimmering*)*-transition* hypothesis? We tested the respective prediction that trigger sites (*ts*) at the nest surface (where shimmering waves had been generated) show higher flickering activity than the alternative non-trigger sites (*nts*). We measured the flickering activity of *ts*- and *nts*-surface bees from two experimental nests, before and after the colony had been aroused by a dummy wasp. Arousal increased rate and intensity of the flickering activity of both *ts*- and *nts* cohorts (P < 0.05), whereby the flickering intensity of *ts*-bees were higher than that of *nts*-bees (P < 0.05). Under arousal, the colonies also increased the number of flickering-active *ts*- and *nts*-cohorts (P < 0.05). This provides evidence that cohorts which are specialist at launching shimmering waves are found across the quiescent nest zone. It also proves that arousal may reinforce the responsiveness of quiescent curtain bees for participating in shimmering, practically by recruiting additional trigger site bees for expanding repetition of rate and intensity of shimmering waves. This finding confirms the *fs-transition* hypothesis and constitutes evidence that flickering is part of a basal colony-intrinsic information system. Furthermore, the findings disprove that the muscle activity associated with *flickering* would heat up the surface bees. Hence, surface bees are not actively contributing to thermoregulation.

## 1. Introduction

Giant Honey Bees (*Apis dorsata*) are famous for being the most dangerous stinging insect on earth [1,2,3,4,5]. However, they are also known for traditional honey hunting and they generate fascination in the scientific community for the multifaceted collective behaviors of their colonies [4,6,7]. Giant Honey Bees are social insects with open-nesting behavior [8,9,10]; they attach their nest with single central semicircular combs beneath tree branches, rock spurs or house balconies [6,10] and orient one of their nest planes to the direction of the sun [9]. They provide unique opportunities to investigate sophisticated collective behaviors which are otherwise, in particular in cave-nesting honeybees (for example in the Western Honey Bee, *A. mellifera*), cryptic for the observer. Giant Honey Bees display swarm behavior in migration [11,12,13,14], reproduction [15], and defense [4,5,16]. A striking example of collective defense in Giant Honey Bees is shimmering [1,2,3,7,10,17; movie 1]. Shimmering is accomplished mostly by mid-aged Giant Honey Bees at the quiescent zones of the nest surface [18] and may therefore constitute a prominent example of division of labor in honeybees [19,20]. Its seemingly wave-like visual pattern repels predators such as wasps, which are the major threat of Giant Honey Bees [7,10]. 

In addition to this highly coordinated shimmering, Giant Honey Bees also display flickering [4,7,21,22] in various behavioral contexts, which represent a collective trait associated with abdomen flipping movements or the movements being singular actions. Flickering displays a diffuse stochastic activity, which can evolve into transient quiescence or shimmering [4]. It seems from visual observation that flickering surface bees flip their abdomens independently, each bee with a different and unsynchronized flickering rate and intensity compared to its neighbor. Up to now, the phenomenon has only been observed in Giant Honey Bees (*Apis dorsata*, *A. laboriosa*) and is only roughly described and termed in literature as *dorso-ventral abdomen flipping* [21,22]. We prefer the term *flickering* over the use of (*dorso-ventral*) *abdominal flipping* since the latter also encompasses a series of other activities in Giant Honey Bees. For instance, Giant Honey Bees use abdominal flipping to divert water droplets away from the nest after exposure to rain [4], to fan the nest [7,9,23,24,25], and to perform waggle and tremble dances [26,27]. It is also the main behavioral component of shimmering [28], and is observed in rearing up [16,29]. Rearing up behaviors typically occur following arousal by either mechanical vibrations of the nest or by sudden approaches of birds, big game, and humans in proximity of the nest. 

Visual observations suggest that flickering does not occur strictly stochastically. Rather, it appears that some areas of the nest surface display activities with higher rates and intensities, which suggests a semi-stochastic or semi-synchronous aspect to flickering. However, we do not know the extent to which the flipping movements in flickering are controlled by random processes or carried over the nest surface in a certain order. It is also unknown whether flickering is affected by the ability of Giant Honey Bees to either modify the rate and strength of their collective behaviors in response to a rising threat, as documented for shimmering waves [30], or to recruit defenders [7,31]. 

In this contribution, we aim to determine under which conditions and to which extent flickering shows random distribution over a patterning that is more lumped in space and time. A deeper understanding of the flickering behavior should come from simultaneously studying properties of the nest surface associated with the generation of shimmering waves. We already know that shimmering waves are initiated by cohorts of bees at specific trigger sites at the nest surface, which also control the propagation of shimmering in response to threatening cues in a wavelike pattern [28]. Parental shimmering waves are generated or triggered (or re-triggered, producing daughter waves) primarily through visual pathways [10,17] while wave propagation is predominantly mechanoceptively controlled [30] and mediated by bucket-bridging strategies from one surface bee to the adjacent one [17]. 

It seems that both modes of social coordination of a Giant Honey Bee colony, *i.e.*, flickering and shimmering, are subject to modification under threat [7]. In particular, the control of intensity and rate of flickering and shimmering often coincide, which allow us to propose that while the colony shifts from the flickering mode to shimmering, it brings the colony’s intrinsic coordination from stochasticity to synchrony. This is subject of the *flickering-shimmering transition* hypothesis. Consequently, we monitored Giant Honey Bee colonies over longer intervals under two arousal conditions, (a) when the colony was quiescent or only occasionally disturbed by natural cues, and (b) when the colony was repetitively exposed to a dummy wasp. We then focused on one of the main potential aspects of the interrelationship between flickering and shimmering and compared the expression of flickering patterns with respect to the trigger sites (*ts*) and non-trigger sites (*nts*) which are, per definition, associated with shimmering. The *flickering-shimmering transition* hypothesis could be accepted under the following two conditions: (a) if the flickering activity of *ts* would be significantly more strongly affected by an external threat than those of *nts*. This would mean that the flickering activity correlates with the arousal state of the surface bees positioned at the *ts*, which is the key condition for generating shimmering waves; furthermore (b), if the flickering activity of *nts* also grades, although less, with the arousal state of the colony. This would mean that *nts* switch to trigger mode. This would increase the numbers of *ts* recruiting generator agents for intensifying strength and rate of shimmering which finally lowers the threshold levels for defensiveness. 

## 2. Material and Methods

### 2.1. Study Site and Experimental Nests

**Figure 1:**
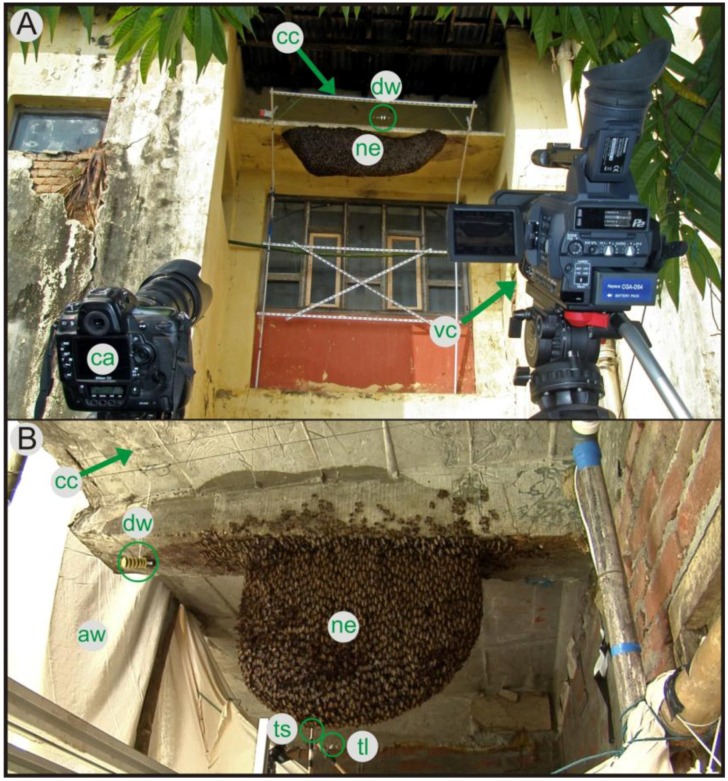
Setup of field stations (Chitwan, Nepal). Experimental nests (**A**: *expN_1_*, **B**: *expN_2_*) were aroused by a dummy wasp (*dw*), which was presented by a computer-controlled cable-car device (*cc*) at constant velocity (v = 0.1−0.5 m/s) in front of the Giant Honey Bee nests (*ne*). The nests were filmed with a high-definition video camera (*vc*) [not seen in B, resolution: 1280 × 720 px, 50 Hz] and a photo camera (*ca*) [not seen in B,6048 × 4032 px, 1 Hz]. Trigger light (*tl*) [not seen in A] was used for the synchronization of HD camera and additional sensory equipment including a thermo sensor (*ts*) [not seen in A] for measuring the ambient temperature and a white awning (*aw*) for screening the experimental setup from direct sunlight (used only in B).

The experiments were conducted with two Giant Honey Bee (*Apis dorsata*) colonies in Chitwan (Nepal). The experimental nest I (*expN_1_*) was located at the Institute of Agriculture and Animal Science (Tribhuvan University, Kathmandu) in Rampur (Chitwan, Nepal) and observations were made in February 2009. There, about 100 fully developed nests co-existed in a radius of two kilometers, which all migrated away three weeks later [14]. The *expN_1_* measured 173 × 59 cm (horizontal × vertical) and possessed honey cells located to its upper left side and at the concave-shaped area to its right (Figure 1A). This spatial disposition indicates that amalgamation of two neighboring nests took place some months ago. The experimental nest II (*expN_2_*) was located at a hotel site in Sauraha (Chitwan National Park) and observations were made in November 2010. This nest was estimated to be approximately three weeks old. The nest had a hemicyclic form (83 × 60 cm) and was attached to balks of concrete of a balcony of the hotel (Figure 1B). Another small queenless colony existed in the surroundings and an additional colony arrived during the course of our experimental session. In the first days of our stay (*i.e.*, on October 28 and 29, 2010) the comb was only covered by a single layer of honeybees. However, after two days, the colony achieved a multi-layer coverage because young bees had progressively hatched. 

### 2.2. Video Recording

The experimental nests were filmed with a high definition video camera (Panasonic HVX 200, resolution: 1280 × 720 px). For the *expN_1_*, the camera was placed 8 m away from the southbound side of the nest and the recordings were made with a resolution of 6 px/cm (Figure 1A). For *expN_2_*, the distance between camera and nest was 1.5 m and the recordings were made with a resolution of 8 px/cm. Both working distances provided an undistorted view of the whole nest and were also sufficient to keep the colony undisturbed, as both colonies accepted the camera as an additional landmark.

### 2.3. Infrared Recording

Infrared thermography [29,31,32] was only used on *expN_2_*. The *expN_2_* was synchronously filmed with an infrared camera (Flir A320; 9 Hz) and the high definition video camera. The cameras covered the whole nest at a nearly perpendicular angle. Long-term infrared monitoring allowed for the detection of the heating-up of bees.

### 2.4. Dummy Wasp Stimulation

We aroused the colonies with a dummy wasp [23]. The dummy wasp was presented by a miniature computer-controlled cable car and was moved with constant velocity (variable between 0.1–0.5 m/s) along a horizontal cable of 20 cm in front of the nests near their attachment zones (Figure 1A,B). For the *expN_1_*, we used the southbound side of the nest (Figure 1A) for the stimulation with the dummy wasp. This stimulation method was chosen to mimic a free-flying wasp scanning in front of the nest.

### 2.5. Observation Session

**Figure 2:**
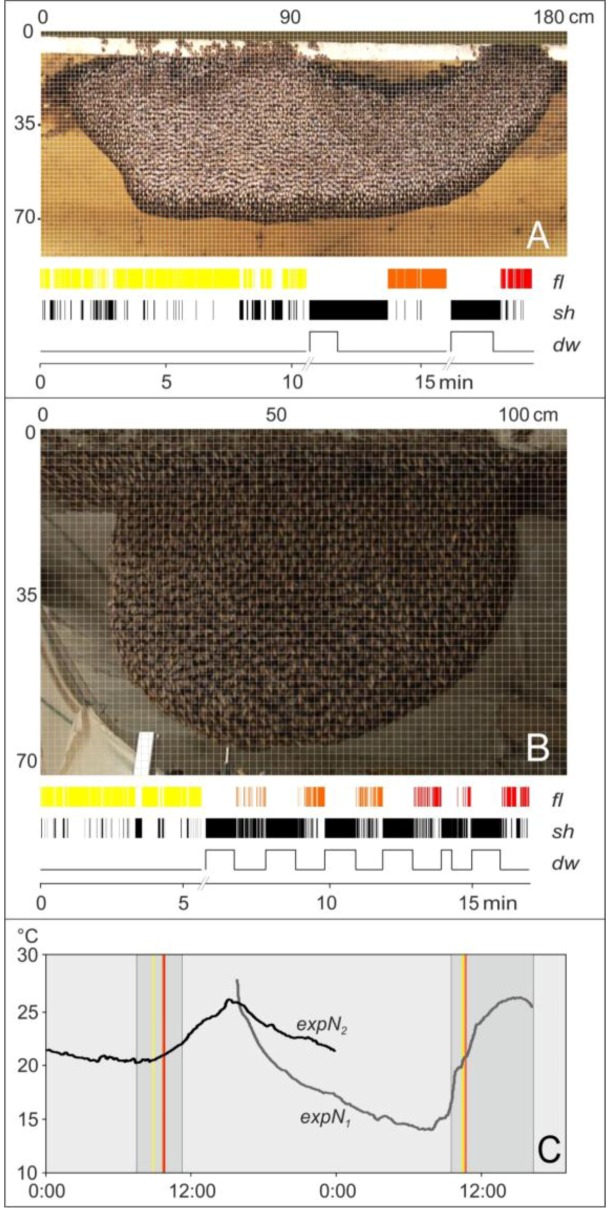
Time scheduling of experiments (**A**: *expN_1_*, **B**: *expN_2_*). The superimposed grids (123 × 53 squares in *expN_1_* and 73 × 48 squares in *expN_2_*) allowed for spatial and temporal analysis of motion (flickering and shimmering) with respect to the repetition rate and intensity of flickering at trigger sites (*ts*) and non-trigger sites (*nts*). A single test square (1.5 × 1.5 cm) corresponds to the area occupied in the image by individual bees at the nest surface and was subject to discrete (test-square-based) statistics. Abscissas and ordinates of the grids are scaled in centimeter. Event diagrams: (*fl*) flickering in *preP* phase [yellow], *postP_1_* phase(s) [orange], and *postP_2_* phase(s) [red]; (*sh*) shimmering waves [black]; (*dw*) presentation of the dummy wasp. The upward lines indicate presentation of the dummy wasp in front of the nest (number of presentations: 2 and 6 for *expN_1_* and *expN_2_*, respectively). (**C**) Typical temperature profiles (*expN_1_*: grey dotted line, *expN_2_*: black-dotted line) measured at the time of the experiment. The filming sessions are identified by grey areas (*expN_1_*: from 9:30 am–4:15 pm on February 5, 2009, *expN_2_*, from 7:30 am–11:15 am on November 16, 2010) and data collection for flickering and shimmering (colored bars give the starting times of the single sessions operating under the following ambient temperatures; *expN_1_*: 19.7 °C; *expN_2_*: 20.4 °C).

We monitored the *expN_1_* and *expN_2_* for three and ten days starting from 10:00 am and 7:00 am until sunset, respectively (Figure 2). At the beginning of each observation session, the test colony was in a quiescent mode (*non-arousal* state) with rare shimmering waves which were provoked by natural stimuli such as birds and insects. Therefore, even at this stage of the experiment, before the dummy wasp was presented, we were able to observe flickering under two conditions, i.e. under *non-arousal* and *arousal*.

We then selected time intervals for the analysis of flickering events (the detailed scheduling is given for both nests in Figure 2C). In the *preP*-phases (marked yellow in Figure 2), the flickering activity was monitored under undisturbed conditions for a total of 12.95 min (e*xpN_1_*: 8.09 min, e*xpN_2_*: 4.86 min). After short pauses in the recording session (e*xpN_1_*: 4.15 min, e*xpN_2_*: 1.00 min), the dummy wasp was presented twice to the *expN_1_* and six times to the *expN*_2_ (see tracking line *dw* in Figure 2A,B).

### 2.6. Experimental Sessions

The performance of the nests was determined by three factors: (i) by the “natural” level of quiescence of the colonies at the start of the experiments; (ii) by the arousal state provoked by repetitive presentation of the dummy wasp; and (iii) by the non-arousal state between each presentation session (Figure 2A,B). In the pre-presentation (*preP*) phase, it was ensured that the colony was calm and undisturbed. For that, the experimental colony was checked for any momentary arousal state. Signs of arousal included a tendency for shimmering without external threat and both the contraction and the elongation [4,31] of the bee curtain (indicating that the colony was ready to release flying guards). The time to recovery after a disturbance depended on the kind of threat. A Giant Honey Bee colony needs tens of minutes to calm down after a raid by either blue-bearded bee-eaters (*Nyctyornis athertoni*) [5] or Oriental Honey-buzzards (*Pernis ptilorhynchus*) [23] while the colony practically recovers within tens of seconds after a wasp attack [10]. The presentations of the dummy wasp (*P*-phase) were launched after the colony had returned to a low-arousal level. Subsequently, the colonies calmed down again. In case of the *expN_1_*, the post-presentation (*postP*) phase was taken to start after the small, “self”-provoked shimmering waves (see above) were not observed for a period of at least 15 s. In the case of *expN_2_*, the *P*-phase lasted for approximately one minute and the *postP* phase started after a pause of one minute. This sequence of *preP-, P*- and *postP*-phases was repeated 6 times. The dummy wasp was presented in several runs individually lasting between 66–100 s and 21–60 s for *expN_1_* (Figure 2A,C) and e*xpN_2_* (Figure 2B,C), respectively.

### 2.7. Assessment of Motion Activities

**Figure 3:**
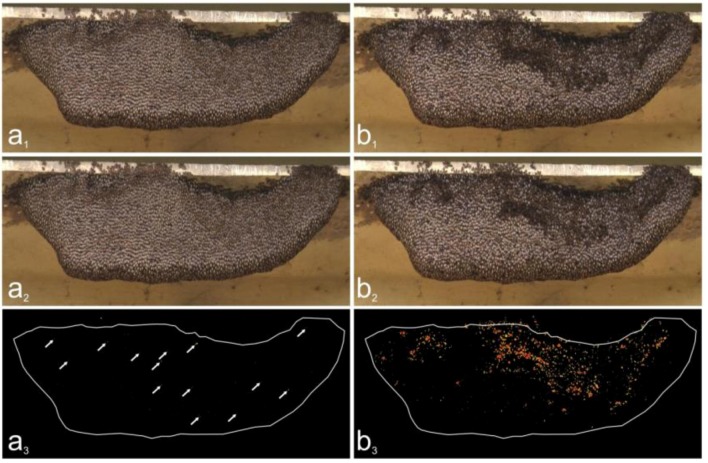
Detection of motion of Giant Honey Bees by image analysis for *expN_1_*. (**a**) Flickering in *preP*-phase (see also movie 1) and (**b**) shimmering in first *P*-phase (see also movie 2). The first two images (**a_1_** and **a_2_**, **b_1_** and **b_2_**) are frames (*f_1_,f_2_*) successively recorded by the HD camera (50 fps). The third image (**a_3_**, **b_3_**) is a differential image (*f_2_-f_1_*) obtained by pixel-operated subtraction and subsequent segmentation (see Methods section). The pseudocolorized patches indicate changes in pixel luminance and indicates bees’ motion activities, with *red* as strong, *orange* as medium, and *yellow* as weak. The white lines give the nest’s contour. Note that during quiescence (as in *preP*-phase), flickering incidents are rare and are seen as singular patches marked by *arrows* (**a_3_**).

Selected video sessions were processed by Avid X-press-Pro Software to fragment single frames and transform them into jpg-format [28]. In total, we analyzed over 20 min of video sequences at 25 fps for *expN_1_* and over 17 min at 50 fps for *expN_2_*, corresponding to approximately 29,000 and 50,000 images, respectively. The images were processed with the Image-Pro and Media Cybernetics programs to detect and quantify movements of single bees at the nest surface [30]. Movements that produce changes in pixel luminance (Δ*lum*) can be identified by image analysis following pixel-operated subtraction and segmentation of the differences between two subsequent images into black-and-white areas (Figure 3). Detectable movements include positional changes in the horizontal and vertical directions and positional changes of the bees’ body parts. A value of Δ*lum* = 0 represents the state of motionlessness and Δ*lum* = 255 the maximum attainable intensity of motion.

### 2.8. Definition of Test Grids

For a quantitative analysis of area-specific motion activity, we have superimposed an assessment grid over the images of the nests (Figure 2A,B). The unit length was set to 15 mm as it corresponds to the size of individual bees at the surface of the nest. The grid possessed 123 × 53 squares in e*xpN_1_* and 73 × 48 in *expN_2_* (horizontal × vertical), corresponding to 184 × 80 cm and 110 × 72 cm areas, respectively.

### 2.9. Assessment of Trigger and Non-trigger Sites

Two categories of nest cohorts were distinguished with respect to the generation of shimmering waves. The trigger sites (*ts*) that generated either parental or daughter shimmering waves in the observation sessions were identified by retrograde inspection of the video films [28]. The locations of *ts*-bees were determined interactively by semi-automated assessment (Image-Pro, Media Cybernetics) of the x- and y-positions of the thoraces, utilizing raw video material combined with difference images (Figure 3). All other sites were termed non-trigger sites (*nts*). By definition, all parental and daughter waves have been launched by *ts*-bees, at least during the span of the observation sessions.

### 2.10. Assessment of Motion Activities at Trigger Sites

**Figure 4:**
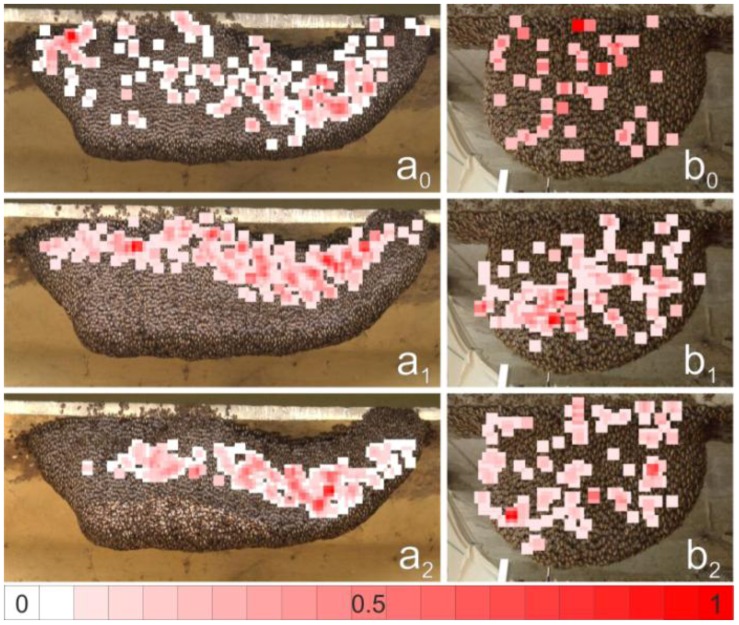
Mapping of *ts* manually detected in the *expN_1_* (**a**) and *expN_2_* (**b**). Test squares where *ts* were identified are colored on a scale from white to red. White squares indicate cases where only one shimmering wave was identified throughout the experimental phase (N_ts_ = 1.0). Red squares (*lum_R_* = 255) indicate the maximum number of shimmering waves detected over the observation interval. The occurrence of shimmering events was scaled separately for each experimental phase. In the *preP*-phase (**a_0_**, **b_0_**), the nests were quiescent and generally undisturbed, with only few shimmering waves provoked by natural cues. In *postP_1-2_*-phases (**a_1‑2_**, **b_1‑2_**), the spatial distribution of *ts* differs between *expN_1_* and *expN_2_*.

Large parental waves often consisted of a series of daughter waves (movie 1) and the trigger activity could extend across single squares of the test grid because it is mostly formed by trigger cohorts [28]. Therefore, we defined *ts* not only by locating the relevant test square (termed *source* square) but rather used a more complex definition; the *ts* corresponded to the positional coordinate of the source square which had been detected manually, but was added up by coordinates of the eight test squares encircling the *source* square. Consequentially, we assigned the trigger activity value 1.0 to nine test squares representing a *ts*. In total, for both experimental colonies, we collected 1453 trigger events from 130 parental shimmering waves and pooled the trigger activity values with respect to their coordinates. The trigger activity values were scaled relative to the maximal sum of trigger activity values assessed in the observation interval (in number of trigger events per test square per reference time interval). The results, plotted in Figure 4, form nuclei and zones with gradual intensity patterns.

### 2.11. Identification of Flickering Activity

**Figure 5:**
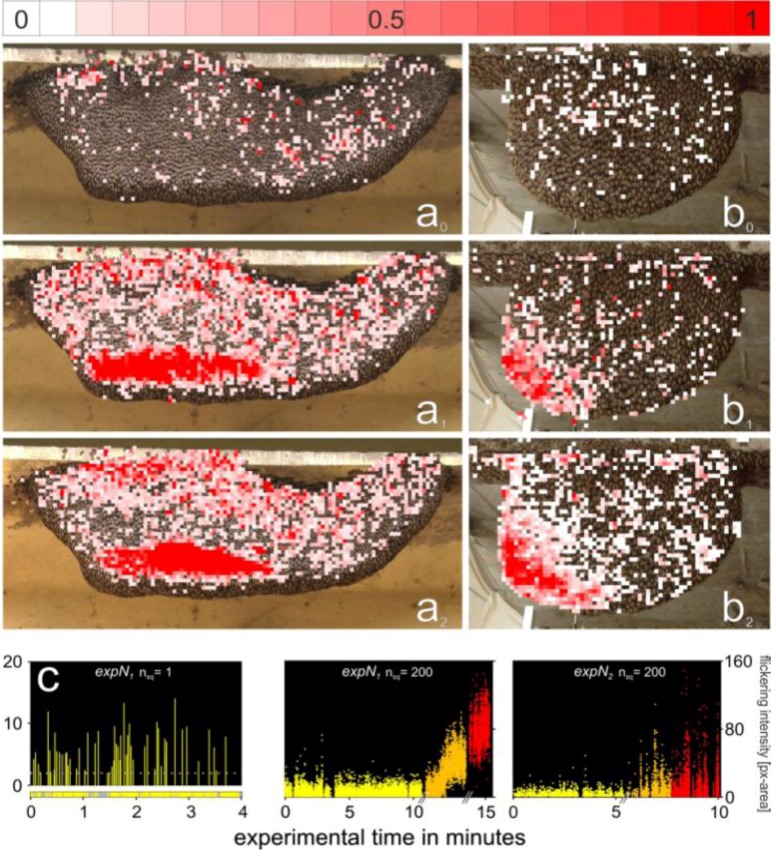
Mapping of flickering activity as evaluated by image analysis in the *expN_1_* (**a**) and *expN_2_* (**b**). Test squares where flickering events were identified are colored on a scale from white to red. White squares indicate cases where a single flickering event was identified, while red squares (*lum_R_* = 255) indicate the maximum number of flickeringevents (flickering repetition rate) detected over the observation interval. Flickering activity was scaled separately for each experimental phase. In the *preP*-phase (**a_0_**, **b_0_**), the nests were quiescent and generally undisturbed, with only few flickering-active test squares of low *flickering* rates. In the *postP_1-2_*-phases (**a_1‑2_**, **b_1‑2_**), the flickering rates increased compared to the *preP*-phase. The spatial distribution of flickering incidences in *postP_1-2_*-phases differs between *expN_1_* and *expN_2_*. Note that the high-motion areas seen at the bottom left sides of the nests (a_1-2_ and b_1-2_) represent mouth zones. These were not considered in the analysis of flickering behavior. (**c**) Typical plot of the flickering incidents recorded in a single test square (n_sq_ = 1) over 240 s in the *preP* phase (shown for *expN_1_*, see also movies 2 and 4). The fine yellow bars represent individual flickering events, the intensity of which is reflected by the height of the bar. The dotted grey line represents the detection threshold for flickering events. Yellow bars in the event diagram (bottom) indicate the phases when data collection for flickering was possible. Grey bars in the event diagram (bottom) indicate the phases when shimmering waves occurred and when detection of *flickering* was not possible. Distribution of flickering incidents with time showing statistical increase of flickering intensity with arousal for *expN_1_* (**d**) and *expN_2_* (**e**). The flickering intensity (px-areas) of the 200 (n_sq_ = 200) most active test squares (each represented as a dot) was plotted for a given recorded frame. Frames were recorded during the *preP-* (yellow) and the *postP_1,2_-*phases (orange and red). The mouth zones were excluded from this analysis.

In difference images, we detected all types of motions of surface bees. The automated differentiation of flickering from other motion categories such as shimmering, walking, dancing or flying, was only possible using interval filters. For the specification of such filter properties, we first identified flickering events and determined their critical duration. We then assessed the changes in luminance (Δ*lum*) generated by selected sample bees over 100 s. A typical *flickering* event was defined for a selected sample bee as having a motion value that exceeded the threshold strength of Δ*lum* > 2.0 for up to three frames (*i.e.*, 60 ms) and was typically repeated after more than one second. In the left diagram of Figure 5c, the motion values collected from a single test square over 4 minutes are monitored. Using our filter, other background activities, such as activities in convection holes (where fanning bees are positioned [33] and produced motion patterns with their wings lasting more than three subsequent frames), could be easily eliminated. Essential interval filters have been applied to allow the identifying and plotting of only-flickering events. However, the filter was unable to discriminate flickering from the many movements occurring in the mouth zones. Flickering events occurring in this area were rather identified by manual inspection and excluded from further automated analyses.

### 2.12. Spatial Distribution of Trigger Sites of Shimmering Waves

We identified the *ts*-bees and determined their image-based x- and y-coordinates sequentially in the shimmering sessions (line *sh* in Figure 2A,B) of the experimental phases (*preP*, *P_i_ & postP_i_*). The incidence of shimmering events at *ts* of parental or daughter waves was plotted into the assessment grids (Figure 2 and Figure 4) in relative scale.

### 2.13. Assessment of the Spatial Distribution of the Flickering Intensity

Each flicker event detected was assigned to its respective test square (Figure 5). For each test square, the total activity level was plotted against (i) the flickering rate (*i.e.*, number of flicker events per min) and (ii) the intensity of flickering (*i.e.*, the total motion strength in the test squares per reference time interval *t_ref_* calculated as _f_*I* = Σ Δlum/t_ref_). As the flickering intensity can be transformed to area of pixels /s, this measure also allows a scaling of the flickering activity: the number of flickering-active bees per second and per reference area of the nest. These data have been collected separately for both the *ts* and *nts* areas and for both the *expN_1_* and *expN_2_*. We further compiled the flickering intensity values according to 30 rate classes (with steps of 0.03 s^−1^). Finally, the means and mean errors of flickering intensities of both cohorts (*ts* and *nts*) were calculated, offering the possibility to compare flickering activities for two nests of different arousal or age conditions. We further verified for systemic differences in flickering intensity _f_*I* between *ts-* and *nts-*cohorts. To do so, we quantified the difference of arithmetical mean of intensity between the two cohorts for each rate class (Δ _f_*I* = _f_*I*_ts_ − _f_*I*_nts_). We sorted the results whether Δ_f_*I* was positive (*i.e.*, _f_*I*_ts_ > _f_*I*_nts_) or negative (*i.e.*, _f_*I*_ts_ < _f_*I*_nts_).

## 3. Results

### 3.1. Observation

In the morning, at the start of the observation session, the ambient temperature was typically 14 °C, with misty conditions in February. During the day, temperatures could climb up to 26 °C in the midday bright sunshine (Figure 2C). The mouth zones [1,10] of the experimental nests were not established in the morning (Figure 2A,B). Only later during the day, after the mist had disappeared, the foragers departed from the nest and the mouth zone became non-*quiescent*, and grew to become 20% of the nest size. The mouth zones were located at the bottom left and at the left side of the *expN_1_* and *expN_2_*, respectively (Figure 5).

### 3.2. Assessment of Flickering and Shimmering Data in the Experimental Phases

At the beginning of each observation session, the test colony was in a quiescent, undisturbed mode (*non-arousal* state), with rare flight and dancing bustles. From time to time, shimmering waves were provoked by natural stimuli, such as birds (e.g., sparrows and pigeons for e*xpN_1_*) and insects (e.g., *Apis cerana* and wasp specimens for e*xpN_2_*). 

In the *preP*-phases, the flickering activity was monitored under undisturbed conditions. During this time, we detected 47 shimmering waves (*expN_1_*: 36, *expN_2_:* 11) that lasted for a total of 3.24 min (Table 1). During the *P*-phase, a series of shimmering waves occurred, indicating that the colonies were aroused by the dummy. After the dummy disappeared, *i.e.*, in the *postP*-phases, the shimmering waves decreased in repetition rate (see the *sh* plots in Figure 2A,B) and intensity. The complementary color-coded *fl*-plots (Figure 2A,B) give the time windows (*expN_1_:* 8.78 min, *expN_2_:* 5.63 min) of the *postP*-phases during which flickering activity was monitored.

**Table 1:** Phases of flickering experiment. *expN_1_, expN_2_:* experimental nests; *expPhase*: experimental phases under evaluation; *preP*: pre-presentation phase; *P_1&2_*: presentation phases, corresponding to periods when dummy wasp was presented; *postP_1&2_*, post-presentation phases, corresponding to periods after the dummy wasp was presented; *N_ts_*: number of trigger sites; *N_waves_*: number of parental shimmering waves selected for evaluation; *relF_ts+nts_*, relative number of flickering-active sites (*ts*+*nts*); *relF_ts_*: of flickering-active trigger sites; *relF_nts_*: of flickering-active non-trigger sites

Nest site	*expPhase*	*N_waves_*	*N_ts_*	*expPhase*	*relF_ts+nts_*	*relF_ts_*	*relF_nts_*
expN_1_	*preP*	36	322	*preP*	0.40	0.18	0.22
	*P_1_*+*postP_1_*	26	325	*postP_1_*	0.86	0.28	0.58
	*P_2_*+*postP_2_*	13	258	*postP_2_*	0.88	0.33	0.55
expN_2_	*preP*	11	61	*preP*	0.34	0.08	0.26
	*P_1_*+*postP_1_*	27	276	*postP_1_*	0.24	0.13	0.11
	*P_2_*+*postP_2_*	17	211	*postP_2_*	0.73	0.26	0.47
*Total numbers*		*130*	*1453*				

### 3.3. Spatial Distribution of Trigger Sites of Shimmering Waves

Shimmering events at *ts* were plotted into the assessment grids (Figure 4) in relative scale. In the *preP* phases, all 47 parental shimmering waves were considered for the analysis of 383 trigger sites (Table 1). During the *P_1_*-, *P_2_*-, and the subsequent *postP_1_-* and *postP_2_*-phases, 25% of the shimmering waves were considered; amounting to 1453 trigger sites (Table 1).

For *expN_1_*, the *preP*-phase *ts* were evenly distributed over the whole nest surface, except for a lesser occurrence in the bottom-left (Figure 4a_0_). Minutes (indicated by the curve) later, the mouth zone developed in that area (Figure 2A), after the morning mist cleared away and the sun shone onto the nest. The regions with highest *ts* were found on the upper left side of the nest and in the concave region to the right side. These *ts* positions changed during the course of the experiment. During the *P_1_*- and *postP_1_*-phases (and also during the *P_2_-* and *post P_2_*-phases) the *ts* positions were distributed on the upper side of the nest, along the trajectory of the dummy wasp. In the *postP_2_*-phase (Figure 4a_2_), the previous *ts* activities located in the upper left nest area disappeared, but were preserved in the concave region to the right side.

The summarized distribution of *ts* of e*xpN_2_* (Figure 4b) gives a different picture, because in all experimental phases, the *ts* were dispersed over the whole nest surface. The main reason for this homogeneous distribution presumably was the smaller nest size, which allowed the dummy wasp to affect the whole nest. In the *preP* phase (Figure 4b_0_), the most active *ts* only showed an incidence of four events over the observation period, because of the low number of naturally provoked shimmering waves (Table 1). However, during the dummy wasp presentations, the number of shimmering waves was high and the number of *ts* increased from the *preP*-phase to the *postP_1_*- and *postP_2_*-phases. The *ts* were distributed in a slightly diagonal band spanning the lower left to the upper right side. The highest numbers of *ts* were found on the upper left side of the nest, where the mouth zone appeared later in the day (Figure 4b_1‑2_). 

### 3.4. Assessment of Flickering Activity

Figure 5 summarizes the flickering activity of the e*xpN_1_* and e*xpN_2_* surface bees during the *preP*- and the *postP_i_*-phases. In the *preP*-phase, the rate of occurrence and the intensity of the flickering were very low (Figure 5a_0_,b_0_, c*_expN1_* [n_sq_ = 1]). The presentation of the dummy wasp dramatically changed the distribution of aroused surface bees and increased the number of flickering incidents. It is to be noted that the mouth zones developed minutes after the recording of the *preP*-phase and before the start of the *postP_1_*-phase, as seen by the areas of high activity. The locomotory activities have been plotted only to illustrate the location of the mouth zone. After the *P_1_*-phases, both nests displayed flickering in the absence of locomotory activity outside the mouth zone, in particular on the upper side of the nest where the dummy wasp passed. Interestingly, the flickering rate of occurrence is astonishingly low in the girdle neighboring the mouth zone (Figure 5a_1-2_), where *ts*-bees were not found (Figure 4a_1-2_). 

**Figure 6:**
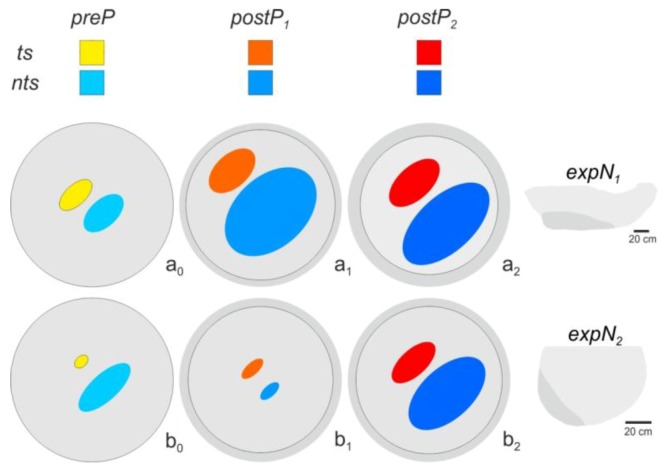
Schematic representation of the changes in proportion of flickering-active trigger sites (*ts*) and non-trigger sites (*nts*) for *expN_1_* (**a**) and *expN_2_* (**b**) observed during the course of experimentation (*preP-*phase: **a_0_**, **b_0_**, *postP_1-2_*-phases: **a_1-2_**, **b_1-2_**) [see Table 1]. The grey areas together represent the relative nest sizes. The *bright grey* circles indicate quiescent zones in which flickering activity was potentially monitored. The *dark grey* rims represent mouth zones (see sketches of the nests to the far right for positioning of the mouth zones in the nests). The mouth zones developed and increased in size over the course of the experiment. The ellipses indicate either the flickering rate of *ts*-areas recorded during the *preP-* (yellow) and the *postP_1,2_-*phases (orange and red) or the flickering rate of *nts*-areas recorded during the *preP-* (light blue) and the *postP_1,2_-*phases (azure and dark blue).

Figure 6 schematizes the development of the two experimental nests during the course of the experiments with respect to the relative number of flickering-active *ts*- and *nts*-bees (Table 1). During the *preP*-phases (Figure 6a_0_,b_0_), the area of flickering-active bees represented a third of both monitored nest surfaces (*expN_1_*: *relF_ts+nts_* = 39.63%, *expN_2_*: *relF_ts+nts_* = 33.67%; Table 1). The relative number of *nts*-bees displaying *flickering* was 22.18% (with 100% being the number of test squares covering the nest area), which is more than the number of flickering-active *ts-*test squares (*relF_nts_* = 17.45%). In *expN_1_*, the number of flickering-active *ts*- and *nts-*test squares increased in the *postP_1_*-phase (*relF_ts+nts_* = 86.42%, *relF_ts_* = 28.54%, *relF_nts_* = 57.88%; Table 1 and Figure 6a_1_) and maintained similar proportions in the *postP_2_*-phase. In *expN_2_*, the number of flickering-active *ts-*test squares increased while that of *nts*-test squares decreased during the *postP_1_*-phase (*relF_ts+nts_* = 23.52%, *relF_ts_* = 12.45%, *relF_nts_* = 11.06%; Table 1 and Figure 6a_1_), but both categories increased to proportions comparable to *expN_1_* in the *postP_2_*-phase. With one exception (*postP*-phase in *expN_2_*), the number of flickering-active *nts*-bees was higher than that of flickering-active *ts*-bees. 

### 3.5. Rate and Intensity Characteristics of Flickering

**Figure 7:**
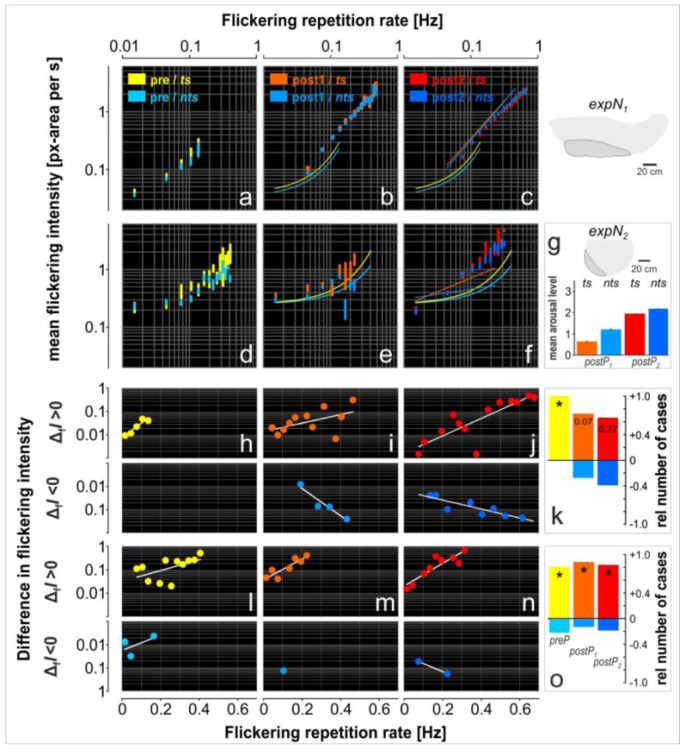
Correlation between *flickering* rate and mean *flickering* intensity observed for *expN_1_* (**a-c**) and *expN_2_* (**d-f**) during the course of experimentation (*preP-*phase: **a**, **d**; *postP_1-2_*-phases: **b**, **c** and **d**, **e**). Flickering activities were measured in *ts-*areas (yellow, orange, and red) and *nts-*areas (light blue, azure, and dark blue). The vertical bars represent the mean ± SEM (the arithmetical means are not explicitly denoted) while the curves are the regressions functions best fitting the results (equations are given in the results section). (**g**) Histogram explaining why the six *postP*-phases in *expN_2_* were treated as two groups (*postP_1_, postP_2_*): the arousal level (flickering repetition rate) strongly increased from *postP_orig3_* (*ts*: from 0.6402 ± 0.0368, n = 234; *nts*: 1.2116 ± 0.0694, n = 271) to *postP_orig4_*(*ts*: 1.9575 ± 0.0268, n = 1018; *nts*: 2.1825 ± 0.0240, n = 1425); similarly also, the spatial flickering rate increased from *postP_orig3 _* to *postP_orig4_* (not shown in Figure 7, see also Results section 3.4.). (**h-j**, **l-n**) Plots of the differences in flickering intensity (ΔI_ts‑nts_) per flickering rate class. The data were sorted into two groups whether the intensity difference was positive (ΔI_ts‑nts_ > 0, upper graphs) or negative (ΔI_ts‑nts_ < 0, lower graphs). Intensity differences were measured in *ts-*areas (yellow, orange, and red) and *nts-*areas (light blue, azure, and dark blue). The curves give the regression functions best fitting the distribution of the difference mean values (refer to Results section for details). (**k**, **o**) Distributions of the *relative numbers of cases* of both groups, the cases of *group_1_* are plotted in positive; the cases of *group_2_* in negative ordinate values. The stars indicate significant differences between the number of positive and negative incidents (P < 0.05). The numeric values inside the columns are the significance level showing a difference between the positive and negative groups.

We used three subsets of parameters to characterize *flickering* as collective behavior. First, rate and intensity (see Methods) were used to examine flickering with respect to non-arousal and arousal. Both parameters can be plotted into the test grid (e.g., Figure 5 shows flickering intensity) to analyze, in second place, the flickering activities of *ts-* and *nts*-bees at the nest surface. Third, the state of arousal was analyzed with respect to the occurrence and the repetition rate of shimmering waves. The arousal state evolved from quiescence to alertness by natural stimuli or by experimental presentation of a dummy wasp. Figure 7 presents the summary of the flickering behavior analysis for the two experimental nests under three arousal states (*preP*, *postP_1-2_*). 

In the rate-intensity plots (Figure 7a-f), the values of flickering intensity were compiled according to 30 rate classes that varied by increments of 0.03 Hz (the total number of events was 6519 for *expN_1_* and 3504 for *expN_2_*). The mean intensity and SEM were transformed into pixel area per s (see Methods). This parameter is suited for comparing the behavior of the two experimental nests, despite their different size and history of arousal.

In the *preP*-phases (Figure 7a and d), the flickering behavior was characterized by rates below 0.4 Hz (*expN_1_*: 0.135 Hz, *expN_2_*: 0.405 Hz) and intensities below 2.0 pixels per s (*expN_1_*: 0.27, *expN_2_*: 2.03). The *ts-* and *nts-*curves can be expressed by exponential functions (y = a. e^b.x^) with the following parameters: *expN_1_* [*ts*]: a = 0.037, b = 15.457, R² = 0.98; *expN_1_* [*nts*]: a = 0.0325, b = 14.583, R² = 0.97; *expN_2_* [*ts*]: a = 0.2485, b = 5.2793, R² = 0.94; *expN_2_* [*nts*]: a = 0.2501, b = 3.8059, R² = 0.80. The curves indicate that, for both categories (*ts, nts*), the more intense the *flickering*, the more frequent the rate becomes (P < 0.001; Spearman correlation test). Figure 7h-n show the differences in flickering intensities between *ts-* and *nts-*cohorts. The positive results were plotted in the upper graphs while the negative results were plotted in the lowers graphs of Figure 7h-j (*expN_1_*) and Figure 7l-n (*expN_2_*) (see Table 2 for regression functions). Finally, the difference between the number of positive and negative cases per arousal condition was analyzed by χ²-test, which showed that the intensity of the *ts*-cohorts in the *preP*-phases was significantly higher than for *nts*-cohorts in most of the rate classes (P < 0.05, χ²-test, see yellow bars in Figure 7k and o).

**Table 2:** Regression functions and coefficients of determination of both cohorts (*ts* and *nts*) for both experimental nests in all experimental phases. Original data are plotted in Figure 7h-j and Figure 7l-n.

*Nest site*	*expPhase*	*ts*	*nts*
*expN_1_*	*preP*	*y = 0,0046e^19,37x^*	*-*
		*R² = 0,8952 *	
	*P_1_+postP_1_*	*y = 0,0102e^5,6643x^*	*y = 0,0035e^18,121x^*
		*R² = 0,2706 *	*R² = 0,9126 *
	*P_2_+postP_2_*	*y = 0,0016e^8,5925x^*	*y = 0,0163e^4,383x^*
		*R² = 0,6911 *	*R² = 0,7555 *
*expN_2_*	*preP*	*y = 0,0328e^7,1152x^*	*y = 0,0148e^-10,05x^*
		*R² = 0,2964 *	*R² = 0,3423 *
	*P_1_+postP_1_*	*y = 0,0389e^13,702x^*	*-*
		*R² = 0,7962 *	
	*P_2_+postP_2_*	*y = 0,0167e^14,98x^*	*y = 0,0281e^10,795x^*
		*R² = 0,8067 *	*R² = 1 *

For *expN_2_*, only two *postP*-phases were considered out of the six sessions of dummy wasp presentation (Figure 2B). This is because the arousal level of the colony (Figure 7g) significantly increased after the *P_3_*-phase, going from 0.64 ± 0.04 to 1.96 ± 0.03 (mean ± SEMs) for *ts*-zones and from 1.21 ± 0.07 to 2.18 ± 0.02 for *nts*-zones (3-fold and 2-fold increase, respectively). Simultaneously, the numbers of flickering-active test squares increased, going from 33 (*P_3_*) to 441 (*P_4_*) for *ts*-areas and from 34 (*P_3_*) to 573 (*P_4_*) for *nts*-areas. Therefore, it seems that the colony had drastically changed its arousal state only after the *P_3_*-phase.

In the *postP_1-2_*-phases, the rate-intensity curves of the *expN_1_ ts*- and *nts*-cohorts shifted to higher intensity and rate values compared to the *preP*-phase curves (Figure 7b,c), with the maximum intensity value going from 0.27 in *preP*-phase to 2.79 in *postP_1_*-phase, and the maximum rate going from 0.135 Hz in *preP*-phase to 0.40 Hz in *postP_1_*-phase. The results of the *postP_2_*-phase are similar to those of the *postP_1_*-phase. The *expN_2_* evolved differently; for this nest, the flickering activity did not significantly change between the *preP*- and *postP_1_-*phases with respect to both intensity and rate (Figure 7d,e). However, a shift to higher values similar to that observed in *expN_1_* occurred in the *postP_2_-*phase, especially with respect to rate (Figure 7f).

The differences in flickering intensity between *ts-* and *nts-*cohorts (Δ_f_*I* = _f_*I*_ts_ − _f_*I*_nts_) in the *postP*-phases were similar in proportion as in the *preP*-phase (Figure 7k,o). Furthermore, the flickering intensities of the *ts*-cohorts were higher than for the *nts*-cohorts in nearly every rate class. This tendency was less pronounced in *expN_1_* (*postP_1_*: P = 0.07, *postP_2_*: P = 0.27) compared to *expN_2_* (*postP_1-2_*: P < 0.05). 

## 4. Discussion

### 4.1. Purposes of Flickering in Giant Honey Bees

The adaptive significance of the *flickering* behavior in Giant Honey Bees has only been vaguely speculated [21] and has yet to be investigated in detail. Nevertheless, from years of experimental work and personal field observations, we are able to say that *flickering* appears to depend on some environmental and colony-intrinsic factors. We know that abdominal flipping correlates, in general, with the release of Nasonov pheromone, as observed for both *Apis mellifera* during fanning either at the hive or at a food source (“Sterzeln” [34,35,36,37]) and *Apis dorsata* during shimmering [38]. In both fanning and shimmering, the last inter-tergital gaps open up, exposing the Nasonov glands and releasing Nasonov. Nasonov is a social pheromone, common to *Apis* species, indicating to the colony members to come to a location identified as either the home nest entrance [34] or a source of water or food [35,39]. It may also communicate to either stay together in a reproduction swarm [36,37] or, specifically for Giant Honey Bees [38] and *Apis cerana* [40], to participate in shimmering. 

### 4.2. Proximate Flickering Hypotheses (Woyke *et al.* 2004)

The *flickering* activity was reported to decrease in number and rate with increasing ambient temperature during the day [21]. A higher percentage of *flickering* surface *A. dorsata* bees was found at 18 °C compared to 20 °C (12.5% *vs*. 7.8%, respectively). Moreover, the flickering rate was higher at 14 °C compared to 18 °C (0.48 Hz *vs*. 0.26 Hz, respectively, n = 26 *A. dorsata* nests). Similar results have been observed in *A. laboriosa* (n = 71 nests). Thus, the authors hypothesized that *flickering* is a response of the colony to low ambient temperature (*flickering-correlates-with-low-temperature* hypothesis). Furthermore, the authors speculated that the muscular activity associated with the act of *flickering* would heat up the bees located at the surface of the bee curtain (*flickering-heats-the-body* hypothesis). 

In our experiments, the recorded mean ambient temperature in the preliminary non-arousal phase was 19.7 °C and 20.4 °C for the *expN_1_* and *expN_2_*, respectively. During this phase, the *expN_1_* colony showed higher rates of *flickeringF* than the *expN_2_* colony (*expN_1_*: *relF_ts+nts_* = 0.3963; *expN_2_*: 0.3367; see also Table 1 and Figure 7), supporting the *flickering-correlates-with-low-temperature* hypothesis. However, a more detailed analysis of the results revealed that only the *ts*-cohorts were more active at lower temperature (*relF_ts_* = 0.1745 and 0.0790 for *expN_1_* and *expN_2_*, respectively, P ≤ 0.05). An opposite trend was observed for the *nts*-cohorts (*relF_nts_* = 0.2218 and 0.2576 for *expN_1_* and *expN_2_*, respectively, P < 0.05). Hence, although we investigated only two nests, our overall results support the *flickering-correlates-with-low-temperature* hypothesis of Woyke *et al*. (2004), at least in this respect, but only for *ts*-cohorts. However, it is important to note that both nests had significantly (P ≤ 0.05; χ²-test) increased their *flickering* behavior under arousal (Table 1; Figure 7). Possibly, and this must be kept open by our findings, this effect is much stronger than the modulation by ambient temperature. 

Infrared recordings convincingly showed that abdominal movements do not alter the body temperature of the agents, both during shimmering (movie 3) and flickering (movie 4) [refer to Methods section for resolution of infrared camera]. Thus, our results do not support the *flickering-heats-the-body* hypothesis of Woyke *et al.* (2004) and further indicate that surface Giant Honey Bees do not actively contribute to the thermoregulation of the nest [41,42] by flickering activities. 

### 4.3. Does the Colony-Intrinsic State induce Diversity in Flickering Activity?

The flickering activities of the two experimental nests differed in some aspects. Firstly, as described above, the *expN_1_* showed a significantly higher spatial flickering rate than the *expN_2_*, especially with respect to *ts* areas (Figure 6 and Table 1)*.* While this aspect is in agreement with the *flickering-rate-correlates-with-ambient-temperature* hypothesis [21], the differences between *expN_1 _* and *expN_2_* displayed in Figure 6 and Figure 7a-f cannot be explained by this hypothesis. The experiments with *expN_1_* and *expN_2_* took place at comparable times and under similar ambient temperatures. Therefore, a plausible explanation for the differences observed between both nests is that specific colony-intrinsic states resulting from, for instance, the season, may influence the flickering activity of the nests. The *expN_1_* monitored at the end of February, when the colony was already quite old. The colony still had a brood of bees and was performing well, with rich honey and pollen stores. Some weeks afterwards, however, the colony departed from this site. By contrast, the *expN_2_* was monitored at the beginning of the dry season (*i.e.*, early November), only some weeks after the colony had arrived at this nesting site. The queen had started to lay eggs and the first young bees had hatched. 

Although the present study does not provide conclusive evidence that colony-intrinsic states affect the control of flickering activity, it nonetheless documents potential characteristics of the colonies that may influence flickering activity. An aspect that can be discussed here is the obvious influence of queen pheromones and juvenile hormones [43,44,45] on the control of the aggression potential of the honey bees. Older bees have higher juvenile hormone levels [46,47,48,49,50] and are more aggressive than younger bees [51,52]. Bees with a higher juvenile hormone level exhibit a quicker response to alarm pheromones [49]. The proportion of bees acting as guards also increases with a higher juvenile hormone level [53], and it is known that the juvenile hormone titers vary seasonally in bees [52]. These aspects could eventually be considered to explain shifts in the proportion of trigger and non-trigger sites in shimmering and the enhancement of the flickering repetition rate over weeks or months in Giant Honey Bees. 

### 4.4. Ultimate Flickering Hypotheses

In this paper, we proposed that the readiness for flickering is affected by the workload of those surface bees that are specifically skilled for generating shimmering waves. Because of their role as leaders in initiating shimmering waves [28], *ts*-cohorts of surface bees would respond more vigilantly under arousal by flickering compared to *nts*-cohorts. We supported this *shimmering-drives-flickering* hypothesis in two experimental nests by first identifying sites responsible for generating shimmering waves and, second, studying the flickering behavior for both cohorts (*i.e.*, *ts*- and *nts*-cohorts) by measuring flickering rate and intensity. The flickering intensity of *ts-*cohorts was generally higher than that of *nts-*cohorts (P < 0.05; χ²-test) in both experimental nests (Figure 7h-n), confirming the *shimmering-drives-flickering* hypothesis. This finding has two further implications. Firstly, this finding indicates that it should theoretically be possible to predict the location of *ts* even before shimmering is provoked by arousal. Secondly, the finding proves a further aspect of division of labor [28,54] in Giant Honey Bees, *i.e.*, that cohorts of specialists are positioned across the nest surface in the quiescent areas peripheral to the mouth zone in order to respond to arousal by increasing flickering rate and by initiating shimmering waves.

We also questioned whether the flickering behavior depended on colony-intrinsic properties, such as arousal history, which is addressed by the *arousal-drives-flickering* hypothesis. For that, we analyzed the arousal state of the colonies by quantifying the number of shimmering episodes that occurred prior to the time intervals when flickering was exclusively displayed. In these pre-arousal (*preP*) phases, the number of shimmering episodes was significantly lower (P < 0.001; t-test) than in post-arousal (*postP_1‑2_*) phases and that was true for both experimental nests. This increase in flickering occurrence concerned both flickering-active *ts-* and *nts-*cohorts (Figure 7a-f), with the *ts-*cohorts showing higher flickering intensity than the *nts-*cohorts (Figure 7i-k, m-o). Under arousal, the number of both cohorts increased (Figure 6a_0_–a_2_, b_0_‑b_2_; Table 1), which documents firstly, that flickering-active cohorts of surface bees augment under arousal and, secondly, Giant Honey Bee colonies recruit generator cohorts (*ts* bees) under arousal as specialists to amplify the subsequent shimmering waves. Finally, recruitment of such generator cohorts requires either persistence or repetition of the threatening signal. 

### 4.5. Flickering as a Mass Recruitment Process

Flickering-active bees typically hang seemingly motionless with the exception of flickering movements. In this way, the bees produce pheromonal, visual, and mechanoceptive signals continuously for hours. Flickering activities apparently stimulate two sensory pathways in the nest mates. Firstly, the abdominal flipping movements produce mechanical cues that undergo strong spatial attenuation and are therefore only of local significance. Secondly, flickering is likely to produce chemical cues, with the last inter-tergital gaps opening up and releasing the social Nasonov pheromone during abdominal flips [38]. Pheromone clouds spread relatively slowly, but would after some time affect the nest mates over a wider range. 

Thus, the signaling coverage achieved with abdominal movements is limited. In the same fashion, signalers like waggle dancers only reach a few post-dancers while fanning bees that disperse Nasonov (“Sterzeln”) are only able to send their message to those bees arriving nearby. The only way to broaden the coverage of any singularly produced signal is mass recruitment of emitters. This is what happens in *flickering* through mechanical, possibly visual, and pheromonal pathways. In combination, these sensory components can indeed benefit a colony by allowing the creation of a growing network of trigger cohorts. It is possible that the concept of “modulatory communication signals”, which is used by *Apis mellifera* for colony-intrinsic communication [45], may also be applied by Giant Honey Bees for flickering. In “modulatory communication signals”, honeybee agents are distributed over the whole hive and produce series of vibration signals over tens of minutes. These agents produce rapid dorso-ventral movements and stay in contact with recipients via the forelegs. Analogously in Giant Honey Bees, an increase in flickering activity of single agents, along with the associated mechanoceptive impulses and pheromones produced, could induce a positive feedback situation to massively recruit surface bees. An increase in intensity and occurrence of flickering may influence the behavior of previously quiescent neighbors that are, in turn, in contact with other neighbors via their legs, in particular by raising their responsiveness to shimmering. 

In social groups, only a small proportion of informed individuals are required [55] to achieve accuracy in controlling the behavioral status of the whole group. The larger the group, the smaller is the proportion of informed individuals needed to guide the group [55]. Our results support this proposition since under quiescent conditions, only a minority of flickering surface bees “suffices” to maintain the colony state. However, under arousal, the colony mobilizes the collective flickering activity by increasing the number of participating agents, by enhancing their flickering rate and intensity, and by enhancing the number of agents capable of initializing shimmering waves. 

Similar aspects of mass recruitment are known among ants [56,57] and termites [58], which not only possess a pheromone mass communication system, but also recruit colony mates via mechanoceptive signals [59,60]. In particular, the substrate vibrations of *Messor capitatus* (Formicidae) are important for communication, but are not essential components of ant recruitment behavior [61]. 

Thus, our findings provide new insights into the complex social behavior of Giant Honey Bee colonies and present quantitative proof that flickering and shimmering are closely related. Flickering of Giant Honey Bees has a modulating role on the generation of shimmering waves and therefore impacts on the defense behavior of Giant Honey Bees. This can be proposed because a higher arousal state of the colony provokes both higher rate and intensity of flickering and higher rate and intensity of shimmering waves.

## 5. Conclusions

Flickering involves abdomen flipping of single bees and presumably stimulates visual, mechanoceptive, and pheromonal signaling pathways. The results of this study suggest that flickering occurs in preparation for shimmering. We were able to show that the trigger sites had higher flickering activity than non-trigger sites. We also have evidence that trigger site bees, which are specialists at initiating shimmering waves, are found across the quiescent zone and that arousal reinforces the receptiveness of quiescent curtain bees for participating in shimmering. This results in the recruitment of additional trigger bees to increase the rate and intensity of shimmering waves. Both modes of social coordination, flickering and shimmering, are subject to modification under threat. Finally, *flickering* is part of a basal colony-intrinsic information system. 

## Supplementary Material

***Movie 1.*** Shimmering behavior in Giant Honey Bees. The movie shows the *expN_1_*, which was attached to the roof of a residential house in Chitwan, Nepal. The movie was recorded with a HD camera at a frame rate of 25 Hz. Above the nest was a black-and-white striped computer controlled dummy wasp, itself mounted on a cable-car device. The dummy wasp provoked shimmering waves. The mouth zone is still at a nascent stage.

***Movie 2.*** Flickering behavior in Giant Honey Bees. The movie shows *expN_1_* during the non-arousal phase, *i.e.*, before the stimulation started. The nest, including the mouth zone, was quiescent and mostly undisturbed, with only a few small shimmering waves provoked by natural cues (not shown). The spatial distribution of flickering behavior is clearly visible. The higher flickering rates are observed in the upper and concave region to the left side of the nest.

***Movie 3.*** Infrared movie of shimmering behavior in Giant Honey Bees. Detailed illustration of abdominal flipping bees at the *expN_2_* during shimmering. Left, HD-image; right, infrared image (in color palette rain900) in temperature range of 20 °C – 40 °C. Infrared recordings convincingly showed that abdominal movements do not alter the body temperature of the surface of the bees during shimmering. Due to the disparity in frame rate (50 Hz and 9 Hz for HD and IR, respectively) the frames are neither congruent nor warp-free. The conversion factor is 9:1. The thorax of a highly flickering active bee was marked by yellow full circle. During the wave episodes, the dot is displaced by a yellow rectangle because thorax detection was difficult. Red patches reflect movements of single bees at the nest surface which were identified by image analysis (see Methods). The dark shadow in some IR frames originates from the cable car dummy. Numbers refer to frame and time (in s) information. 

***Movie 4.*** Infrared movie of flickering behavior in Giant Honey Bees. Detailed illustration of abdominal flipping of bees at the *expN_2_* during flickering. Left, HD-image; right, infrared image. The same bee is marked as in movie 3. Infrared recordings showed that flickering movements do not alter the body temperature of surface bees. During the flickering episodes, the flickering active bee was marked by a yellow rectangle. More details are shown in additional movie 3.

## Supplementary Files

Supplementary File 1
